# Targeting PIKfyve: Chemical Biology and Drug Discovery Insights

**DOI:** 10.1111/cbdd.70395

**Published:** 2026-09-01

**Authors:** Athavan Alias Anand Selvam, Anamika Sharma

**Affiliations:** ^1^ Department of Chemistry Prayoga Institute of Education Research Bengaluru Karnataka India; ^2^ Peptide Science Laboratory, School of Chemistry and Physics University of KwaZulu‐Natal Durban South Africa

## Abstract

PIKfyve inhibitors have evolved from niche probes into powerful tools for studying endolysosomal biology. Although PIKfyve inhibition has revealed important roles in membrane trafficking, autophagy, viral entry, and cancer‐cell vulnerability, clinical translation remains limited by a narrow therapeutic window because disruption of PI(3,5)P_2_ homeostasis causes profound lysosomal dysfunction. Future progress will depend on tuneable or partial inhibitors, improved selectivity, and targeted delivery. PIKfyve therefore provides a useful model for balancing potency, precision, and safety in lipid kinase drug discovery.

## Graphical abstract

PIKfyve inhibitors have progressed from niche chemical probes to powerful tools for interrogating endolysosomal biology, enabled by rapid and robust cellular phenotypes. Compounds such as apilimod establish PIKfyve as a druggable lipid kinase, while agents like vacuolin‐1 underscore the importance of distinguishing true target engagement from phenotypic effects.

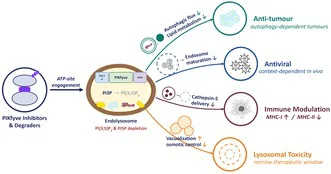

## 1 Introduction and Scope

Phosphoinositide lipid kinases occupy a central position at the interface of membrane biology and signal transduction, yet they have historically lagged protein kinases as therapeutic targets. Early challenges including limited structural information, concerns about selectivity within the ATP‐binding pocket, and the complexity of phosphoinositide signalling networks limited their use as therapeutics (Fruman et al. 2017). However, the clinical success of Class I Phosphoinositide 3‐kinase (PI3K) inhibitors and advances in chemo‐proteomics and structural biology have renewed interest in lipid kinases as druggable enzymes (Balla 2013; Fruman et al. 2017). Within this expanding landscape, phosphoinositide kinase FYVE‐type zinc finger containing (PIKfyve) has emerged as a distinctive and chemically tractable target (Balla 2013). PIKfyve catalyses the phosphorylation of PI3P to generate PI(3,5)P_2_ (a defining phosphoinositide of late endosomes and lysosomes) and contributes to PI5P production—lipids that are uniquely enriched on endolysosomal membranes and are essential for membrane fission, trafficking, and osmotic homeostasis (McCartney et al. 2014; Shisheva 2008; Zolov et al. 2012).

**FIGURE 1:**
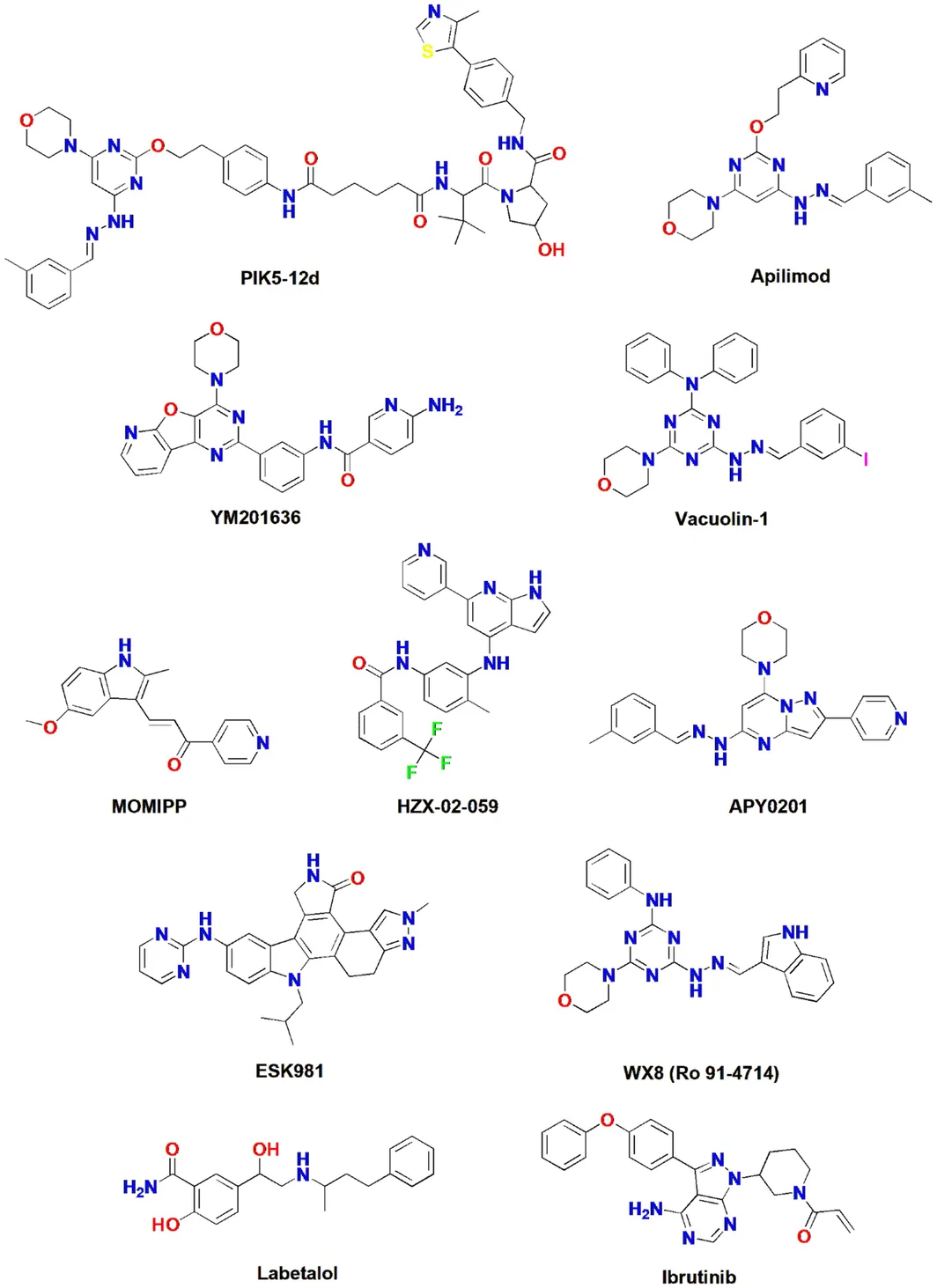
Representative small‐molecule and PROTAC‐based PIKfyve inhibitors discussed in this review: PIK5‐12d (Li et al. 2023); apilimod (Cai et al. 2013); YM201636 (Jefferies et al. 2008); vacuolin‐1 (Sano et al. 2016); MOMIPP (Cho et al. 2018); APY0201 (Hayakawa et al. 2014); HZX‐02‐059 (Feng et al. 2022); ESK981 (Qiao et al. 2021); WX8/Ro 91‐4714 (Roy and DePamphilis 2024; Sharma et al. 2019); ibrutinib and labetalol (Wadje et al. 2025).

Acute pharmacological inhibition of PIKfyve produces rapid and striking cytoplasmic vacuolization across diverse cell types, providing a robust and easily quantifiable cellular phenotype that has accelerated chemical probe development. Disruption of this pathway leads to defects in vesicular transport and lysosomal function, underscoring its central role in cellular homeostasis (Lenk and Meisler 2014; Saffi et al. 2022). A diverse repertoire of small‐molecule probes has been developed to interrogate PIKfyve biology and its therapeutic potential. Some of the small‐molecule inhibitors which have helped establish PIKfyve as a druggable therapeutic target as shown in Figure 1. The first widely used selective inhibitor, apilimod, initially developed as a suppressor of pro‐inflammatory cytokines IL‐12 and IL‐23, and was later shown to directly inhibit PIKfyve with nanomolar potency (Cai et al. 2013; Jefferies et al. 2008). The widely used inhibitors YM201636 and vacuolin‐1 suppress PI(3,5)P_2_ production and disrupt endolysosomal trafficking, lysosomal maturation, and autophagic flux (Jefferies et al. 2008; Sano et al. 2016), whereas apilimod is a potent PIKfyve inhibitor with demonstrated activity in B‐cell malignancies (Cai et al. 2013). APY0201 is a highly selective ATP‐competitive inhibitor that has shown antitumour activity in multiple myeloma and gastric cancer models (Hayakawa et al. 2014). Subsequent studies have broadened the inhibitor landscape to encompass structurally diverse chemotypes, such as triazine‐based compounds, indolyl derivatives, and related heterocyclic scaffolds (Chen et al. 2024; Cho et al. 2018; Roy and DePamphilis 2024; Sharma et al. 2019; Xing et al. 2025). Other chemotypes, including MOMIPP (Cho et al. 2018) and the WX8 (Roy and DePamphilis 2024; Sharma et al. 2019) compound family, induce extensive vacuolation and methuosis or impair lysosomal homeostasis in autophagy‐dependent cancer cells. HZX‐02‐059 combines PIKfyve inhibition with tubulin‐disrupting activity and produces methuosis and cell‐cycle arrest in double‐hit lymphoma (Feng et al. 2022). More recently, the PROTAC PIK5‐12d enabled selective degradation of PIKfyve, producing sustained inhibition of autophagic flux and growth suppression in prostate‐cancer models (Li et al. 2023). Collectively, these chemically and mechanistically distinct agents have established PIKfyve as a tractable regulator of endolysosomal homeostasis, autophagy, and cancer‐cell survival, while also emphasizing the need to distinguish selective PIKfyve engagement from compound‐specific or polypharmacological effects. Reports that approved drugs (e.g., ibrutinib or labetalol) display weak or context‐dependent PIKfyve activity computationally further underscore the need for rigorous target validation (Wadje et al. 2025).

PIKfyve inhibition has demonstrated biological relevance across multiple disease contexts. In oncology, it preferentially affects tumour cells dependent on lysosomal turnover and autophagy, including models of melanoma, multiple myeloma, lymphoma, hepatocellular carcinoma, non‐small‐cell lung cancer, prostate cancer, and pancreatic ductal adenocarcinoma (Chakraborty et al. 2022; Cheng et al. 2025; DoĞan et al. 2021; Feng et al. 2022; Hou et al. 2019; Llorente et al. 2025; Qiao et al. 2021). In liver‐ and lung‐cancer models, YM201636 reduced tumour‐cell proliferation and malignant characteristics while perturbing autophagy‐ and endolysosomal‐associated signalling (DoĞan et al. 2021; Hou et al. 2019). In prostate cancer, ESK981‐mediated PIKfyve inhibition disrupted autophagic flux, promoted CXCL10‐dependent T‐cell recruitment, and enhanced the response to immune‐checkpoint blockade; reproduction of key effects by PIKfyve knockdown and structurally distinct PIKfyve inhibitors strengthened their attribution to PIKfyve engagement rather than to the broader kinase activity of ESK981 (Qiao et al. 2021). PIKfyve inhibition can also limit lysosomal degradation of MHC Class I, thereby increasing its surface expression and enhancing CD8^+^ T‐cell‐mediated tumour‐cell killing (Bao et al. 2023; Qiao et al. 2021). In pancreatic cancer, genetic or pharmacological suppression of PIKfyve reduced PI(3,5)P_2_ and PI5P levels, disrupted lysosomal and autophagic function, altered lipid and cholesterol homeostasis, and inhibited tumour progression. These effects were further enhanced by concurrent inhibition of Kirsten rat sarcoma viral oncogene homolog gene (KRAS) and its downstream mitogen‐activated protein kinase (MAPK) signalling pathway (Cheng et al. 2025). Collectively, these studies demonstrate that convincing PIKfyve target validation requires concordant genetic, biochemical, and pharmacological evidence because vacuolation, impaired autophagic flux, or reduced cell viability alone may represent pathway‐associated phenotypes rather than definitive proof of direct target engagement.

In immune systems, PIKfyve regulates antigen processing and presentation (Baranov et al. 2019), while antiviral studies demonstrate that interfering with endosomal pathways disrupts viral entry and replication (Baker et al. 2023; Logue et al. 2022; Luo et al. 2023). Despite these advances, the medicinal chemistry space for PIKfyve inhibitors remains constrained. Most compounds rely on ATP‐competitive engagement of a conserved kinase fold via heteroaromatic scaffolds. While these achieve high potency, selectivity remains challenging due to the conserved catalytic architecture across lipid kinases (Ikonomov et al. 2019; Wadje et al. 2025). The absence of high‐resolution inhibitor‐bound structures further necessitates reliance on empirical structure–activity relationship (SAR) strategies (Lazaro et al. 2020).

An additional layer of complexity arises from the intracellular context of PIKfyve function. Because the enzyme acts on membrane‐associated substrates, inhibitors must achieve sufficient membrane permeability and intracellular exposure to be effective (Alemany and Morel 2025; Gu et al. 2022). Consequently, biochemical potency does not always translate into cellular efficacy, underscoring the importance of physicochemical optimization. These challenges highlight the need for expanded chemical diversity and refined design strategies to enable selective modulation of PIKfyve activity while maintaining an appropriate balance between potency and cellular tolerability.

This review focuses on the chemical and translational dimensions of PIKfyve modulation. We examine inhibitor discovery strategies, SAR, pharmacological behaviour, and emerging translational liabilities. While PIKfyve inhibition delivers exceptionally strong apparent cellular potency and visually striking phenotypes, it also presents challenges in selectivity, chemical control, durability of response, and therapeutic window, issues that define the frontier of lipid kinase drug discovery.

## 2 PIKfyve Biology Relevant to Drug Discovery

PIKfyve is a large multidomain lipid kinase whose structural organization supports its membrane recruitment, complex assembly, regulation, and catalytic function. The protein contains an *N*‐terminal FYVE domain that binds PI3P and facilitates localization to endosomal membranes, together with central cysteine‐rich and chaperonin‐like CCT regions involved in protein organization and regulation. Its C‐terminal phosphoinositide kinase domain catalyses the ATP‐dependent conversion of PI3P to PI(3,5)P_2_ and represents the principal site targeted by currently characterized ATP‐competitive inhibitors (Lees et al. 2020; Sbrissa et al. 2002; Shisheva 2008). Although the noncatalytic domains and protein–protein interaction surfaces may offer opportunities for future allosteric or complex‐disrupting agents, they have not yet been validated as direct binding sites for established PIKfyve inhibitors.

**FIGURE 2:**
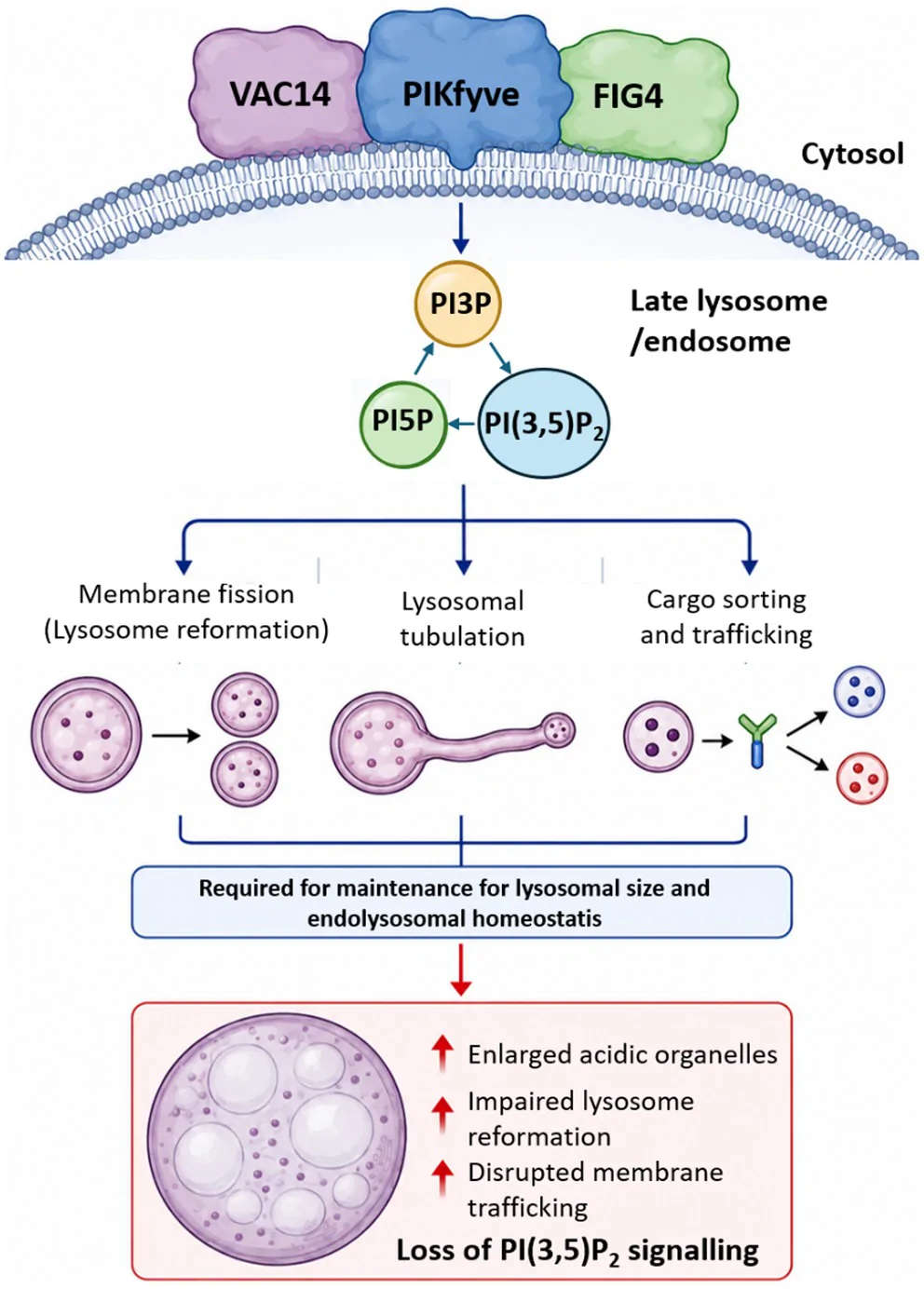
Schematic representation of PIKfyve function and signalling.

PIKfyve regulates endolysosomal function through tightly controlled phosphoinositide turnover in late endosomal and lysosomal compartments. The enzyme functions as part of a heterotrimeric complex comprising the scaffold protein VAC14 and the phosphatase FIG4, which together coordinate the synthesis and turnover of PI(3,5)P_2_ and maintain lipid homeostasis (Kutchukian et al. 2025; Rivero‐Ríos and Weisman 2022; Sbrissa et al. 2007) as shown in Figure 2. PI(3,5)P_2_ in turn regulates membrane remodelling on late endosomal and lysosomal membranes, including membrane fission (lysosome reformation), lysosomal tubulation, and cargo sorting (Krishna et al. 2016; Li et al. 2013). These processes maintain lysosomal size and homeostasis, and disruption of PI(3,5)P_2_ signalling results in enlarged acidic organelles due to impaired lysosome reformation (Li et al. 2013). A second drug‐relevant output of the PIKfyve pathway is PI5P. In vivo studies indicate that a substantial proportion of cellular PI5P is PIKfyve‐dependent and that PI(3,5)P_2_ serves as a major precursor for its synthesis thereby linking PIKfyve catalytic activity to PI5P‐regulated membrane‐protein interactions (Rivero‐Ríos and Weisman 2022; Zolov et al. 2012). PI5P can be generated through dephosphorylation of PI(3,5)P_2_ by catalytically active myotubularin‐family 3‐phosphatases, which remove the phosphate group at the 3‐position of the inositol ring. MTMR3 has been directly implicated in this pathway and functions together with PIKfyve in a phosphoinositide‐conversion route in which PI3P is first phosphorylated to PI(3,5)P_2_ and subsequently dephosphorylated to PI5P (Oppelt et al. 2012). Other active myotubularins, including MTM1 and MTMR2, can also hydrolyse PI(3,5)P_2_, although their relative contribution to cellular PI5P production may vary with cell type and subcellular context. On endolysosomal membranes, PIKfyve functions within the VAC14 and FIG4 complex to convert PI3P into PI(3,5)P_2_, which can subsequently contribute to PI5P generation. Accordingly, inhibition of PIKfyve disrupts the coordinated production of both phosphoinositides and perturbs multiple stages of the endosomal‐lysosomal pathway. This disruption commonly produces enlarged vacuolated compartments; however, their molecular identity is context dependent and varies with cell type, inhibitor, exposure duration, and the markers examined, with studies reporting EEA1‐positive endosomal structures, LAMP1‐positive lysosomal compartments, or mixed endolysosomal characteristics (Cheng et al. 2025; Hazeki et al. 2013).

An important consideration in target validation is that genetic impairment and pharmacological inhibition of PIKfyve can produce overlapping cellular effects, including depletion of PI(3,5)P_2_ and PI5P, enlargement of endolysosomal compartments, and disruption of membrane trafficking (Lees et al. 2020). However, they diverge in ways that matter for target selection: (i) genetic disruption (including of complex partners PIKfyve/VAC14/FIG4) (Chow et al. 2007); and (ii) pharmacological inhibition which can reveal therapeutic windows (Kim et al. 2014; Lenk and Meisler 2014; Saffi et al. 2022). However, the consequences of genetic perturbation vary considerably according to gene dosage, tissue specificity, developmental timing, and the nature of the mutation. Complete systemic deletion of PIKfyve in mice causes preimplantation embryonic lethality, whereas heterozygous animals are viable and develop normally, demonstrating that the effects of PIKfyve deficiency depend strongly on the extent of functional loss (Ikonomov et al. 2011). In contrast, tissue‐specific deletion can produce more restricted phenotypes. For example, myeloid‐specific PIKfyve deficiency causes enlarged lysosomal compartments, impaired lysosomal degradation, altered lysosomal protein homeostasis, macrophage accumulation, and systemic inflammation without generalized neurodegeneration or immediate lethality (Min et al. 2019). Partial or heterozygous PIKfyve dysfunction in humans can also produce localized disease rather than a lethal systemic phenotype. Heterozygous PIKfyve variants are associated with François‐Neetens fleck corneal dystrophy, an autosomal dominant disorder characterized predominantly by non‐progressive stromal corneal flecks and generally preserved vision (Kotoulas et al. 2011; Li et al. 2005). These findings demonstrate that PIKfyve loss of function does not produce a uniform phenotype and may range from embryonic lethality to tissue‐restricted abnormalities, depending on residual protein activity and the affected cellular context (Kim et al. 2014; Lenk and Meisler 2014; Saffi et al. 2022).

Genetic disruption of the VAC14 or FIG4 may further produce severe neurological abnormalities because these proteins are required for the organization and regulated activity of the PIKfyve‐VAC14‐FIG4 complex (Lees et al. 2020). Pharmacological inhibition, by contrast, is generally acute, dose dependent, and potentially reversible, allowing partial suppression of PIKfyve activity without necessarily reproducing the developmental and tissue‐specific consequences of lifelong genetic deficiency. Thus, although concordant cellular phenotypes support engagement of the PIKfyve pathway, differences in residual activity, exposure duration, complex stability, and tissue context must be considered when comparing genetic and pharmacological perturbations.

## 3 Structural Features and Druggability

PIKfyve is a multidomain phosphoinositide kinase that integrates a conserved ATP‐dependent catalytic architecture with membrane‐specific regulatory control. Its C‐terminal catalytic domain is predicted to share the conserved structural architecture of phosphoinositide kinases; however, PIKfyve functions primarily on endolysosomal membranes, unlike Class I PI3Ks, which mainly mediate growth‐factor signalling at the plasma membrane (Lees et al. 2020; Sbrissa et al. 2000).

Instead, PIKfyve functions predominantly at late endosomal membranes, where localized lipid composition defines organelle identity (Rivero‐Ríos and Weisman 2022). Structurally, PIKfyve contains an *N*‐terminal FYVE domain that mediates recruitment to PI(3)P‐enriched endosomal membranes, spatially restricting its catalytic activity. Within the *C*‐terminal kinase domain, Lys1831 is a critical residue in the predicted ATP‐binding and catalytic region of PIKfyve. Substitution of this residue abolishes lipid‐kinase activity and prevents the production of PI(3,5)P_2_ and PI5P, leading to pronounced endomembrane enlargement and vacuolation (Ikonomov et al. 2002, 2001; Sbrissa et al. 2000). By contrast, Lys1999 and Lys2000 are located within a putative lipid‐substrate recognition region and appear to influence phosphoinositide specificity rather than ATP binding. These residues provide important mechanistic context for understanding PIKfyve catalytic function and the activity of ATP‐competitive inhibitors. Its C‐terminal kinase domain functions within a multiprotein complex organized by the scaffold VAC14 and containing the PI(3,5)P_2_ phosphatase FIG4 (Ikonomov et al. 2001; Sbrissa et al. 2000). This interdependent assembly ensures dynamic regulation of PtdIns(3,5)P_2_ synthesis and turnover. Disruption of any component destabilizes lipid homeostasis and alters lysosomal morphology, underscoring that PIKfyve functions as part of a coordinated regulatory module rather than an isolated catalytic enzyme. From a druggability perspective, the ATP‐binding cleft of PIKfyve is demonstrably targetable but structurally conserved. Biochemical inhibition across multiple scaffold classes consistently achieves low‐nanomolar potency (Hou et al. 2024). However, selectivity margins relative to other phosphoinositide kinases often range from approximately one order of magnitude to two orders of magnitude, depending on scaffold refinement (Wadje et al. 2025). This limitation reflects conserved hinge geometry and catalytic core architecture shared across lipid kinases, constraining opportunities for discrimination within the ATP‐binding pocket.

Importantly, druggability cannot be evaluated solely by enzymatic inhibition. Because PIKfyve catalyses phosphorylation of a membrane‐embedded lipid substrate, effective inhibitors must access endosomal compartments and achieve sufficient intracellular exposure. Consequently, cellular potency frequently diverges from biochemical affinity, emphasizing the importance of physicochemical tuning alongside catalytic optimization (Alemany and Morel 2025; Gu et al. 2022). These considerations distinguish PIKfyve from cytosolic kinases, where catalytic engagement more directly predicts functional outcome. To date, reported inhibitor strategies primarily rely on ATP‐competitive engagement, and alternative allosteric approaches remain underexplored (Chen et al. 2024; Lazaro et al. 2020). Because high‐resolution structures of PIKfyve bound to inhibitors remain unavailable, inhibitor optimization has relied largely on empirical SAR studies, in which chemical modifications are evaluated experimentally for their effects on potency and selectivity (Lazaro et al. 2020). Notably, in the absence of co‐crystal structures, current understanding of inhibitor binding modes is largely inferred from SAR trends and kinome profiling rather than direct structural evidence. This constraint underscores that druggability in PIKfyve is shaped not only by catalytic tractability but also by limited structural divergence within the conserved kinase fold. PIKfyve presents a catalytically tractable yet structurally constrained ATP‐binding site that permits potent inhibition; however, membrane‐embedded function and regulatory complex integration narrow the margin for selective modulation.

## 4 Chemical Biology of PIKfyve Inhibition

Chemical inhibition of PIKfyve has provided a powerful framework for dissecting how acute perturbation of phosphoinositide metabolism reshapes endolysosomal homeostasis. Across structurally diverse inhibitor classes, suppression of PtdIns(3,5)P_2_ synthesis disrupts membrane fission and late endosomal maturation (Ikonomov et al. 2019; Jefferies et al. 2008). Reversibility studies following YM201636 withdrawal demonstrate restoration of endosomal architecture, confirming that early trafficking defects arise from functional lipid depletion rather than irreversible lysosomal damage (Jefferies et al. 2008). These observations establish PIKfyve as a dynamic regulator of membrane remodelling rather than a constitutive structural determinant. Beyond general trafficking effects, chemical inhibition alters coordinated maturation events within immune compartments. In macrophages, PIKfyve blockade delays PI(3)P turnover on phagosomes and reduces acquisition of lysosomal markers, indicating impaired late‐stage maturation (Kim et al. 2014). In dendritic cells, inhibition interferes with cathepsin S trafficking and attenuates MHC Class II antigen processing (Baranov et al. 2019). This defect is attributed to impaired late endosomal protease delivery and delayed invariant chain processing, thereby limiting efficient peptide loading and CD4^+^ T‐cell activation. These findings support a mechanistic link between PI(3,5)P_2_‐dependent membrane dynamics and adaptive immune regulation. Oncologic systems have revealed how this membrane vulnerability can be therapeutically exploited. In autophagy‐dependent melanoma models, triazine‐based inhibitors such as the WX8 series suppress autophagosome–lysosome fusion and impair degradative flux, resulting in selective cytotoxicity (Chakraborty et al. 2022; Sharma et al. 2019). In multiple myeloma, pharmacologic inhibition activates TFEB‐driven lysosomal transcriptional programs while simultaneously blocking functional autophagy, leading to tumour growth suppression (Gayle et al. 2017). Collectively, these observations suggest that tumour contexts reliant on sustained autophagic flux may exhibit increased susceptibility to catalytic modulation of PIKfyve.

Antiviral investigations further illustrate the context‐dependent consequences of lipid kinase inhibition. In epithelial systems, PIKfyve inhibition reduces replication of multiple RNA viruses (Baker et al. 2023; Luo et al. 2023). Mechanistically, this effect reflects disruption of endosomal maturation and ESCRT‐dependent membrane remodelling required for viral entry and cytoplasmic genome release. However, for certain in vivo infection models, systemic inhibition has been associated with worsened disease severity (Logue et al. 2022). This divergence likely reflects broader perturbation of endosomal function within immune compartments, underscoring the complexity of host‐directed antiviral modulation. A unifying feature across biological contexts is the narrow tolerance of endolysosomal systems to reductions in PtdIns(3,5)P_2_ availability. Modest catalytic suppression can produce amplified trafficking consequences, whereas partial recovery restores membrane dynamics. Consequently, biochemical IC_50_ values alone do not predict phenotypic amplitude; intracellular distribution, exposure duration, and tissue‐specific resilience critically shape biological outcome (Misaghi et al. 2025; Xing et al. 2025). Chemical modality further modifies response durability. ATP‐competitive inhibitors transiently suppress lipid production, permitting restoration following dissociation. In contrast, PROTAC‐derived degraders (PIK5‐12d) induce sustained depletion of PIKfyve protein (Li et al. 2023). Because degradation reduces protein abundance rather than transiently occupying the ATP site, restoration of PI(3,5)P_2_ depends on de novo protein synthesis, altering recovery kinetics and extending functional suppression. The chemical biology of PIKfyve inhibition reveals a context‐sensitive endolysosomal control node. Reversible catalytic modulation enables interrogation of trafficking, immune processing, tumour metabolism, and viral replication. However, the same membrane sensitivity that allows robust phenotypic amplification constrains translational flexibility.

## 5 Chemical Classes and Structure Activity Relationship (SAR) of PIKfyve Inhibitors

The medicinal chemistry development of PIKfyve inhibitors illustrates a recurring theme: catalytic inhibition is readily achievable, whereas selectivity, exposure control, and phenotypic precision require deliberate structural refinement. Although multiple chemotypes reach low‐nanomolar biochemical potency, subtle modifications in peripheral substituents and physicochemical balance markedly influence kinome breadth and endolysosomal response. Comparative analysis across major scaffold classes reveals that affinity alone does not dictate functional outcome; rather, geometry within the conserved ATP‐binding pocket and membrane engagement properties govern translational viability. Substituent‐driven modulation of lipophilicity and polar surface area critically influences lysosomal accumulation and intracellular target engagement. Thus, effective optimization requires balancing catalytic affinity with compartment‐specific exposure rather than relying solely on kinase binding potency. Figure 1 shows various types of PIKfyve kinase inhibitors.

### 5.1 Morpholine‐Containing Pyrimidines and Aminopyrimidines

YM201636 established early proof‐of‐concept that PIKfyve can be inhibited competitively within the ATP‐binding cleft, achieving enzymatic IC_50_ values near 30 nM (Lees et al. 2020; Sbrissa et al. 2007). Selectivity margins over Class I PI3Ks in early series generally ranged from approximately 10–50‐fold, reflecting the conserved architecture of lipid kinase catalytic domains. SAR studies demonstrated that morpholine‐mediated hinge interaction is essential for potency, whereas modifications in adjacent aromatic regions modulate cross‐reactivity and cellular amplitude (Sele et al. 2017). The observed shift between biochemical and cellular potency underscores the importance of membrane accessibility and intracellular exposure in translating catalytic engagement into biological effect. Apilimod (STA‐5326) represents a more refined aminopyrimidine scaffold that achieves subnanomolar enzymatic potency with improved kinome selectivity (Cai et al. 2013; Cheng et al. 2025). However, its strong biochemical activity has not translated into comparable clinical efficacy. Apilimod showed limited benefit in Phase II trials for Crohn's disease and rheumatoid arthritis, and its development has been constrained by low systemic exposure and progressive loss of cellular activity, suggesting suboptimal pharmacokinetic stability and insufficient sustained target engagement in vivo (Ikonomov et al. 2019; Krausz et al. 2012; Sands et al. 2010). Apilimod is proposed to occupy the ATP‐binding pocket of the PIKfyve kinase domain, where Asn1939 contributes to inhibitor recognition. Consistent with this model, the N1939K substitution confers more than 150‐fold resistance to apilimod, providing functional evidence for the involvement of this residue in compound binding (Gayle et al. 2017). Nevertheless, the precise interaction remains to be confirmed by a high‐resolution inhibitor‐bound structure. Thus, although apilimod remains an important proof‐of‐concept inhibitor for selective PIKfyve targeting, its clinical experience highlights the need for analogues with improved exposure, metabolic stability, and tissue distribution.

Structural variation at solvent‐exposed positions influences selectivity margins without substantially altering catalytic affinity, indicating that discrimination among lipid kinases depends primarily on substituent orientation beyond the conserved hinge region. This scaffold exemplifies how steric tuning outside the core ATP‐binding motif can expand selectivity space while preserving potency.

### 5.2 Triazine‐Based Chemotypes

The WX8 triazine series, identified through phenotypic screening in autophagy‐dependent melanoma models, represents a chemically distinct optimization strategy. Optimized analogues demonstrate sub‐10 nM enzymatic inhibition and robust suppression of autophagic flux (Roy and DePamphilis 2024). SAR analysis revealed several hundred‐fold variations in potency depending on substituent orientation within the triazine core, highlighting the sensitivity of catalytic engagement to subtle steric rearrangements. Reversibility studies indicate that lysosomal perturbation induced by these compounds reflects competitive catalytic inhibition rather than irreversible membrane destabilization. However, increasing lipophilicity correlates with heightened nonspecific cytotoxicity, reinforcing that physicochemical balance is essential for preserving selectivity. Across this series, functional autophagy assays were indispensable for distinguishing productive PIKfyve inhibition from generalized membrane stress.

### 5.3 Indolyl‐Derived Scaffolds

Indolyl‐pyridinyl‐propenone derivatives, including MOMIPP analogues, were initially characterized by methuosis‐like phenotypes and later shown to inhibit PIKfyve with nanomolar affinity (Cho et al. 2018). SAR investigations demonstrated that substitutions at specific indole positions can redirect biological outcome from lipid‐kinase–dependent endolysosomal disruption toward microtubule interference and apoptotic pathways. This phenotype divergence underscores the sensitivity of downstream biology to modest alterations in ATP‐pocket orientation and reinforces the necessity of orthogonal validation to confirm catalytic specificity. Subsequent indolyl‐pyrimidinamine scaffolds were developed to improve selectivity while retaining low‐nanomolar enzymatic inhibition (Drewry et al. 2022). These compounds achieve potent catalytic engagement despite lacking classical morpholine hinge motifs, suggesting alternative interaction geometries within the ATP‐binding cleft. Kinome‐wide profiling indicates improved discrimination against related lipid kinases, demonstrating that scaffold diversification can mitigate cross‐reactivity while maintaining affinity.

### 5.4 Dual‐Target and Targeted Degradation Approaches

The azaindole derivative HZX‐02‐059 exemplifies a dual‐target strategy, simultaneously inhibiting PIKfyve and tubulin (Feng et al. 2022). Although producing endolysosomal perturbation consistent with lipid kinase blockade, concurrent cytoskeletal effects complicate mechanistic interpretation, emphasizing the importance of kinase profiling and pathway validation when evaluating multifunctional compounds. A mechanistically distinct modality involves PROTAC‐based degraders (PIK5‐12d) derived from ATP‐competitive warheads (C. Li et al. 2023). These molecules induce low‐nanomolar DC_50_–mediated depletion of PIKfyve protein, resulting in sustained suppression of lipid synthesis. Because degradation eliminates catalytic protein rather than transiently occupying the ATP site, restoration of PI(3,5)P_2_ requires de novo protein synthesis rather than simple inhibitor dissociation. SAR considerations in this framework extend beyond binding affinity to include linker composition, E3 ligase geometry, and ternary complex stability, all of which determine degradation efficiency and temporal durability. Across chemotypes, several consistent principles emerge. First, hinge engagement confers high catalytic potency but increases the risk of lipid kinase cross‐reactivity due to conserved ATP‐binding architecture. Second, substituents extending beyond the conserved hinge region are primary determinants of selectivity. Third, modulation of lipophilicity governs membrane partitioning and functional amplitude. Finally, catalytic affinity must be interpreted in light of kinome profiling and phenotypic validation to ensure selective engagement.

## 6 Context‐Dependent Biological Outcomes of PIKfyve Inhibition

Given its conserved catalytic architecture and narrow tolerance to reductions in PtdIns(3,5)P_2_ availability, inhibition of PIKfyve produces biological outcomes that vary with cellular dependency, exposure duration, and tissue context. While suppression of lipid kinase activity uniformly perturbs endolysosomal trafficking, downstream consequences differ markedly across physiological systems. These context‐dependent responses reflect differential reliance on membrane recycling, autophagic flux, and immune processing pathways. In oncologic models, PIKfyve inhibition reveals a vulnerability in tumors dependent on sustained lysosomal turnover. Autophagy‐reliant melanoma cells exhibit pronounced sensitivity to triazine‐based inhibitors, with disruption of autophagosome–lysosome fusion leading to metabolic stress and selective cytotoxicity (Sharma et al. 2026). Similarly, in multiple myeloma, catalytic suppression activates TFEB‐mediated lysosomal gene transcription while concurrently impairing degradative capacity, resulting in growth inhibition (Hasegawa et al. 2022). Emerging evidence from solid tumour systems further suggests that sensitivity correlates with dependence on lysosomal recycling rather than proliferative signalling alone. Collectively, these findings indicate that tumour susceptibility appears to correlate strongly with reliance on endolysosomal flux and metabolic buffering capacity. In contrast, immune compartments demonstrate a different sensitivity profile. In macrophages and dendritic cells, inhibition of PIKfyve alters phagosome maturation and antigen processing, affecting protease trafficking and MHC Class II presentation (Baranov et al. 2019). Rather than uniformly suppressing immune function, these perturbations modify antigen presentation efficiency and inflammatory signalling in a context‐dependent manner. Such effects underscore that modulation of endosomal lipid composition intersects directly with adaptive immune regulation.

Antiviral studies further illustrate the context‐dependent consequences of PIKfyve inhibition. Consistent with the antiviral mechanism outlined in Section 4, PIKfyve blockade also suppresses replication of several RNA viruses in epithelial cell models (Karim et al. 2025), reinforcing that endosomal integrity is a shared vulnerability across viral families rather than a feature unique to any single system. However, in certain in vivo infection models, systemic inhibition has been associated with exacerbated disease severity (Logue et al. 2022). These contrasting outcomes underscore that host‐directed modulation of membrane trafficking may yield protective or detrimental effects depending on immune integrity, viral burden, and tissue‐specific resilience. Metabolic and organ‐specific systems provide additional nuance. In models of obesity‐associated cardiomyopathy, controlled PIKfyve modulation has been associated with improved mitochondrial function and reduced oxidative stress through SIRT3‐associated pathways (Tronchere et al. 2017). These findings suggest that moderate lipid kinase modulation can recalibrate organelle homeostasis under metabolic stress, rather than simply induce collapse. Across these diverse contexts, a consistent principle emerges whereby biological outcome depends less on the magnitude of catalytic inhibition and more on the system's reliance on continuous endolysosomal flux. Cells with high autophagic or trafficking demand exhibit amplified responses to catalytic suppression, whereas systems with greater membrane redundancy may tolerate partial perturbation. Importantly, reversible inhibition permits recovery of lipid homeostasis, whereas prolonged suppression or protein degradation may shift the balance toward sustained dysfunction.

## 7 Toxicity and Therapeutic Window

Despite strong genetic and pharmacological validation of PIKfyve as a disease‐relevant target, its therapeutic exploitation is constrained by a narrow therapeutic window driven by intrinsic, on‐target toxicity. As a central regulator of endolysosomal homeostasis, PIKfyve controls phosphoinositide flux that underpins membrane trafficking, vesicle maturation, and lysosomal function. Consequently, its inhibition perturbs fundamental cellular processes essential for viability, particularly in cells with high endocytic or autophagic demand (Uwada et al. 2025; Yang et al. 2024).

The dominant toxicity associated with PIKfyve inhibition arises from disruption of endosomal and lysosomal dynamics. Acute inhibition causes rapid enlargement of late endosomes and lysosomes, extensive cytoplasmic vacuolation, impaired cargo trafficking, and defective autophagic flux. Beyond its role in endosomal cargo sorting and recycling, PIKfyve is also required for the resolution of cargo‐containing phagolysosomal vacuole shrinkage and the recovery of nutrients generated through cargo degradation (Krishna et al. 2016). Mechanistically, this process was shown to depend partly on the lysosomal cation channel Transient Receptor Potential Mucolipin 1 (TRPML1), linking the PIKfyve‐PI(3,5)P_2_ axis to lysosomal Ca^2+^ release and membrane‐remodelling events that facilitate vacuole resolution. Accordingly, disruption of PIKfyve or TRPML1 activity impairs the clearance and shrinkage of enlarged phagolysosomal compartments and compromises nutrient recovery. Although these phenotypes serve as robust pharmacodynamic markers, they directly reflect loss of membrane identity and osmotic control (Bonolo de Campos et al. 2026; Karim et al. 2025; Kutchukian et al. 2025; Logue et al. 2022). Critically, these toxicities are dose‐ and cell‐type dependent, defining the narrowness of the therapeutic window. Cells with high vesicular turnover such as neurons, epithelial cells, and immune populations are particularly sensitive, whereas transient or partial inhibition can be tolerated in certain contexts with reversible vacuolation. This creates a sharp threshold between tolerable pathway modulation and irreversible cellular damage, indicating that biological indispensability, rather than off‐target effects, limits safety (Drewry et al. 2022).

Both genetic and pharmacological evidence reinforce this constraint where complete loss of PIKfyve function is incompatible with normal tissue homeostasis, and high levels of inhibition consistently produce dose‐limiting lysosomal toxicity. As a result, PIKfyve does not conform to a “maximal inhibition” paradigm. Instead, its therapeutic window is defined by the ability to finely tune target engagement, where excessive potency without control becomes a liability.

Accordingly, dosing strategies must prioritize controlled, partial, or transient inhibition to remain within a tolerable range. This reframes PIKfyve as a target where precision of modulation outweighs absolute potency, emphasizing that successful drug design must align with the biological limits of the system.

## 8 Conclusions and Outlook

PIKfyve has matured from a chemical curiosity into one of the most extensively validated lipid kinases in endolysosomal biology, owing to the availability of inhibitors that yield rapid, robust, and morphologically striking cellular readouts. Apilimod and structurally distinct scaffolds including the triazine‐based WX8 series, indolyl derivatives, and the PIK5‐12d degrader have collectively established PIKfyve as a chemically addressable node governing membrane trafficking, autophagy, viral entry, and cancer‐cell survival. Compounds such as vacuolin‐1, once considered purely phenotypic tools, are now understood to engage PIKfyve directly, a reassessment that underscores how rigorous chemical and pharmacological validation continues to refine which cellular phenotypes reflect true target engagement. Despite this progress, clinical translation remains constrained: the same acute disruption of PI(3,5)P_2_ homeostasis responsible for these strong phenotypes is difficult to confine spatially or temporally in vivo, so achieving efficacious target engagement without systemic lysosomal toxicity remains the field's central challenge.

Progress in this field will likely depend on advances in medicinal chemistry and delivery, including the development of tuneable or partial inhibitors and targeted delivery systems. More broadly, PIKfyve provides an instructive paradigm for lipid kinase drug discovery. It illustrates both the opportunities afforded by strong, pathway‐defining phenotypes and the challenges of translating these effects into safe and controllable therapeutics. Lessons learned from probe validation to phenotype‐target decoupling and therapeutic window optimization are likely to inform future efforts across the lipid kinase family, where balancing potency with precision will be critical for clinical success.

## Author Contributions

**Athavan Alias Anand Selvam:** writing – review and editing, conceptualization, formal analysis. **Anamika Sharma:** conceptualization, methodology, formal analysis, writing – original draft, writing – review and editing.

## Funding

The authors have nothing to report.

## Conflicts of Interest

The authors declare no conflicts of interest.

## Data Availability

The authors have nothing to report.
